# Trends in the multiple prescriptions of hypnotic drugs in a university outpatient in Japan

**DOI:** 10.1002/npr2.12386

**Published:** 2023-11-09

**Authors:** Takao Kato, Nozomu Kotorii, Motohiro Ozone, Kenta Murotani, Hayato Ohshima, Hiroyuki Mori, Kenjirou Wasano, Hiroshi Hiejima, Mitsunari Habukawa, Naohisa Uchimura

**Affiliations:** ^1^ Department of Neuropsychiatry Kurume University School of Medicine Fukuoka Japan; ^2^ Department of Psychiatry Kato Hospital Kisarazu Japan; ^3^ Department of Psychiatry Kotorii Isahaya Hospital Nagasaki Japan; ^4^ Biostatistics Center Kurume University Fukuoka Japan

**Keywords:** antidepressants, antipsychotics, anxiolytics, hypnotics, insomnia, polypharmacy, psychotropic drugs

## Abstract

**Aims:**

In Japan, the daily dosage of hypnotic drugs for insomnia treatment is increasing year by year, and over‐dependence on treatment with hypnotic drugs is a major problem. This study aimed to examine the factors related to the elimination of prescriptions of three or more hypnotic drugs within 1 year in our clinic.

**Methods:**

We conducted two surveys. Survey ① assessed the frequency of prescriptions of three or more hypnotic drugs by retrospectively reviewing the medical records of all patients who visited general and psychiatric outpatient clinics from January 2013 to March 2019. Survey ② assessed changes in prescriptions of hypnotic and psychotropic drugs within the subsequent year by retrospectively reviewing the medical records of all patients prescribed three or more hypnotic drugs who visited neuropsychiatric outpatient clinics multiple times between April 2013 and March 2019.

**Results:**

The frequency of prescribing three or more hypnotic drugs was six to nine times higher in psychiatry than in other departments. Flunitrazepam and brotizolam were the most common drugs prescribed and had the second lowest discontinuation rate after zolpidem. Conversely, eszopiclone, zopiclone, and suvorexant had the highest discontinuation rates. The success factors for drug reduction were age (odds ratio [OR]: 0.97, *p* < 0.0037), trazodone addition (OR: 12.86, *p* < 0.0194) and number of years of psychiatric experience.

**Conclusions:**

The characteristics and success factors in relation to drug reduction in patients with multiple prescriptions of hypnotic drugs identified in this study may contribute to solving the problem of multiple prescriptions of hypnotic drugs.

## Abstract 2

This study examines the factors related to the elimination of prescriptions of three or more hypnotic drugs within 1 year in our clinic. The success factors for drug reduction were age, trazodone addition and number of years of psychiatric experience.

## Graphical abstract

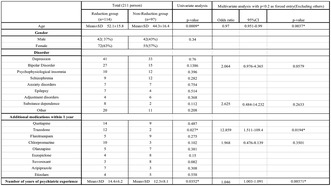

## 1 INTRODUCTION

Insomnia is associated with generalized daytime malaise and difficulty concentrating, which can easily lead to a decline in psychomotor function and quality of life.^1^, ^2^ In addition, insomnia has been shown to be a risk factor for not only physical diseases, such as hypertension and diabetes,^3^, ^4^ but also psychiatric disorders, such as depression.^5^ Therefore, the proper treatment of insomnia is extremely important. However, looking at the actual situation of insomnia treatment in Japan, the daily dosage of hypnotic drugs is increasing year by year,^6^ and there is a major problem of over‐dependence on drug treatment with hypnotic drugs. As a measure to improve this situation, the 2014 revision of medical reimbursements in Japan eliminated continuing psychiatric outpatient support and made it so that guidance fees would not be calculated when more than three hypnotic drugs were included in a single prescription. In addition, the prescription, prescribing, and drug fees were also reduced. Although these measures have resulted in a slight decrease in the rate of prescriptions of five or more hypnotic drugs, from 1.8% before to 0.9% at 6 months after the revision, no change in the downward trend of the rate of multiple prescriptions of two to four hypnotic drugs has been observed since the revision, indicating that the situation has not yet been sufficiently corrected.^7^ In addition, the characteristics of patients with multiple prescriptions of hypnotic drugs and the factors that determine whether they subsequently succeed in reducing their dosage have not yet been examined. Few previous studies have examined the factors that may have contributed to the resolution of poly‐administration of three or more hypnotic drugs in patients on poly‐administration therapy. Therefore, we conducted a retrospective, noninterventional, observational study based on medical records to investigate the factors that help patients prescribed three or more hypnotic drugs to reduce their medications to fewer than three drugs within 1 year at our outpatient clinic.

## 2 METHODS

### 2.1 Participants

The hypnotic drugs investigated in this study were γ‐aminobutyric acid (GABA) receptor agonist sleep medications (eszopiclone, zolpidem, zopiclone, triazolam, flunitrazepam, brotizolam, quazepam, estazolam, rilmazafone, and lormetazepam), ramelteon, and suvorexant, which were used in the hospital as of 2019. First, the change in the frequency of hypnotic prescriptions for three or more drugs in patients diagnosed with insomnia at our outpatient clinic was investigated from April 2013 to March 2019 among all those who visited general departments (all departments except pediatrics and neuropsychiatry) and psychiatric outpatients. Second, prescriptions for the 1 year after a multidrug prescription were also investigated in psychiatric ambulatory patients who were prescribed a hypnotic drug multiple times over any 3‐month period between April 2013 and March 2019 and continued medical examinations for at least 1 year afterwards. In this study, patients who were prescribed hypnotic drugs based on a diagnosis of insomnia in the attending physician's medical record were included in the analysis.

### 2.2 Study design

#### 2.2.1 Study methods

In this study, prescriptions for three or more sleep medications were defined as “multiple prescriptions,” and the ratio of the number of multiple prescriptions for hypnotic drugs to the total number of prescriptions was defined as the “multiple prescription rate.” Changes in the multiple prescription rate in general medicine and psychiatry between April 2013 and March 2019 were calculated by surveying all patients (excluding pediatric patients) who visited the outpatient department during this period and surveying the status of hypnotic drug prescriptions through the electronic medical record system. For those who had been prescribed multiple medications in psychiatry between April 2013 and March 2019, changes in prescriptions during the subsequent 1 year were investigated retrospectively. We also investigated the percentage of attending physicians who had reduced their patients' prescriptions to two or fewer hypnotic drugs within 1 year. To identify factors that would help eliminate the use of multiple hypnotic drugs, patients for whom the number of hypnotic drugs had been reduced to two or fewer within 1 year were classified into the reduction group, and those who had remained on three or more drugs were classified into the non‐reduction group.

#### 2.2.2 Survey items

The primary survey items were as follows: (1) the multiple prescription rate of hypnotic drugs in general medicine and psychiatry, and (2) change in the use of sleeping pills 1 year later among those prescribed multiple sleeping pills in psychiatry. The secondary items were as follows: sex, age, psychiatric disorder, and psychotropic medications added within 1 year (i.e., anxiolytics, antidepressants, and antipsychotics), attending physician, and number of years of psychiatric experience.

### 2.3 Statistical analysis

Statistical analysis was performed to compare items such as age, gender, psychiatric disorders, and psychotropic medications added within 1 year between the reduction and non‐reduction groups. An unpaired *t* test was used to compare normal factors, the Mann–Whitney *U* test to compare non‐normal factors, and the chi‐square test to compare differences in proportions between the two groups. In addition, we assessed the patients' clinical variables using univariate and multivariate logistic regression modeling to determine odds ratios (ORs) and 95% confidence intervals (CIs) to determine whether multiple prescriptions for hypnotic drugs had been eliminated. Variables included in the multivariate models had a *p* ≤ 0.2 in the univariate analysis.^8^ To test the success rate of reducing hypnotic drugs by the number of years of psychiatric experience, we used receiver operating characteristic (ROC) curve analyses. The chi‐square test was used to test differences in the drug reduction ratio between two groups: “sleep specialist or physician specializing in psychopharmacology” and “other psychiatrists.” All data analyses were performed using SAS 9.4 (SAS Institute Inc., Cary, NC, USA) and R version 4.2.1.^9^

## 3 RESULTS

### 3.1 Frequency of prescribing three or more hypnotic drugs inside and outside psychiatry

**FIGURE 1:**
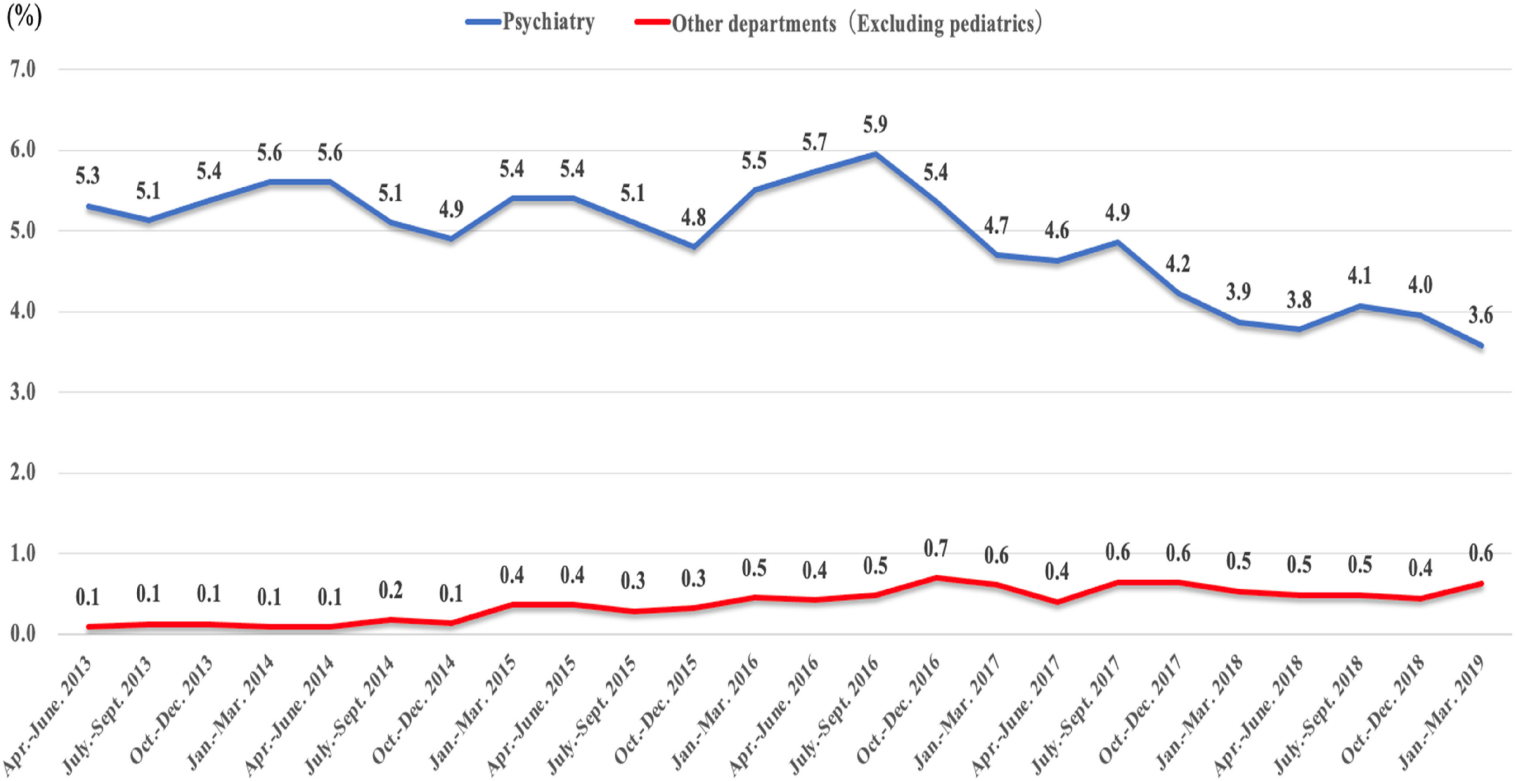
Frequency of prescribing three or more hypnotic drugs inside and outside psychiatry.

Figure 1 shows the results of our primary study on the multiple prescription rate of hypnotic medications in psychiatry and other departments (excluding pediatrics) for the 6‐year period from April 2013. The multiple prescription rate of hypnotic drugs in psychiatry trended downward from 5.1% to 5.4% in 2013, from 4.9% to 5.6% in 2014, from 4.8% to 5.4% in 2015, from 5.4% to 6% in 2016, from 4.2% to 4.9% in 2017, and from 3.6% to 4.1% in 2018, whereas the rate in other departments remained flat, at around 0.4–0.6% in all years.

### 3.2 Characteristics of patients with multiple prescriptions for hypnotic drugs in outpatient psychiatry

#### 3.2.1 Background characteristics of patients with multiple prescriptions for hypnotic drugs in outpatient psychiatry

Table 1 shows the background characteristics (gender, age, and primary psychiatric illness) of patients with multiple prescriptions of hypnotic drugs in a psychiatric outpatient clinic (84 males [39.8%], 127 females [60.2%], mean age ± standard deviation [SD]: 47.9 ± 15.8 years). The most common primary psychiatric illness was depression (*n* = 74), followed by bipolar disorder (*n* = 42), psychophysiological insomnia (*n* = 22), schizophrenia (*n* = 21), and anxiety disorder (*n* = 14).

**TABLE 1:** Backgrounds of patients with multiple prescriptions of hypnotic drugs.

	Overall (*n* = 211)
Age	Mean ± SD 47.9 ± 15.8
*Gender*	
Male	84 (40%)
Female	127 (60%)
*Disorder*	
Depression	74
Bipolar disorder	42
Psychophysiological insomnia	22
Schizophrenia	21
Anxiety disorders	14
Epilepsy	11
Adjustment disorders	10
Substance dependence	10
Other	31
*Additional medications within 1 year*	
Quetiapine	23
Trazodone	14
Flunitrazepam	14
Chlorpromazine	13
Olanzapine	12
Eszopiclone	12
Suvorexant	11
Aripiprazole	10
Etizolam	9
*Number of years of psychiatric experience*	Mean ± SD 13.4 ± 7.2

Abbreviation: SD, standard deviation.

#### 3.2.2 Hypnotic drugs among the prescribed drugs

The highest number of prescriptions among all 211 for three or more hypnotic drugs was 132 (63%) for flunitrazepam, followed by 126 (60%) for brotizolam, 83 (39%) for eszopiclone, 75 (36%) for ramelteon, 63 (30%) for suvorexant, 48 (23%) for zolpidem, and 42 (20%) for zopiclone.

#### 3.2.3 Psychotropic drugs added within 1 year

The most common psychotropic drugs added within 1 year from the day when three or more hypnotic drugs were prescribed for the first time were quetiapine in 23 patients (10.9%), followed by trazodone in 14 (6.6%), flunitrazepam in 14 (6.6%), chlorpromazine in 13 (6.2%), olanzapine and eszopiclone in 12 (5.7%), suvorexant in 11 (5.2%), aripiprazole in 10 (4.7%), and etizolam in 9 (4.3%).

### 3.3 Reduced group and non‐reduced drug groups

Table 2 shows the profiles of the patients in the reduction group, for whom the number of prescribed hypnotic drugs had been reduced to two or fewer within 1 year from the date of the first prescription of three or more hypnotic drugs, and in the non‐reduction group, for whom three or more hypnotic drugs were still being prescribed. The proportion of the reduction group to the total number of patients (reduction rate of hypnotic drugs) was 54%.

**TABLE 2:** Profiles of the reduction and non‐reduction groups.

	Total (211 person)		Univariate analysis	Multivariate analysis with *p* < 0.2 as forced entry (excluding others)		
	Reduction group (*n* = 114)	Non‐reduction group (*n* = 97)	*p*‐Value	Odds ratio	95%CI	*p*‐Value
Age	Mean ± SD 52.1 ± 15.8	Mean ± SD 44.3 ± 16.4	0.0009^a^	0.97	0.951‐0.99	0.0037^a^
*Gender*						
Male	42 (37%)	42 (43%)	0.34			
Female	72 (63%)	55 (57%)				
*Disorder*						
Depression	41	33	0.76			
Bipolar disorder	27	15	0.1386	2.064	0.976–4.365	0.0579
Psychophysiological insomnia	10	12	0.396			
Schizophrenia	9	12	0.282			
Anxiety disorders	7	7	0.754			
Epilepsy	7	4	0.514			
Adjustment disorders	4	6	0.368			
Substance dependence	8	2	0.112	2.625	0.484–14.232	0.2633
Other	20	11	0.208			
*Additional medications within 1 year*						
Quetiapine	14	9	0.487			
Trazodone	12	2	0.027^a^	12.859	1.511‐109.4	0.0194^a^
Flunitrazepam	5	9	0.275			
Chlorpromazine	10	3	0.102	1.968	0.476–8.139	0.3501
Olanzapine	5	7	0.381			
Eszopiclone	4	8	0.15			
Suvorexant	3	8	0.082			
Aripiprazole	7	3	0.308			
Etizolam	4	5	0.558			
*Number of years of psychiatric experience*	Mean ± SD 14.4 ± 6.2	Mean ± SD 12.3 ± 8.1	0.0352^a^	1.046	1.003‐1.091	0.00371^a^

Abbreviations: CI, confidence interval; SD, standard deviation.

^a^ Groups differed significantly at *p* < 0.05.

#### 3.3.1 Age and gender

The mean ages of the patients in the reduction and non‐reduction groups were 52.1 ± 15.8 and 44.3 ± 16.4 years, respectively, indicating a significant difference between the two groups, with younger patients less able to reduce their dose (*p* < 0.0009). On the other hand, no significant difference in the dose reduction rate was seen by gender (*p* < 0.34).

#### 3.3.2 Hypnotic drug reduction rate by psychiatric disorder

The hypnotic drug reduction rates for each psychiatric disorder were 55% (41/74 patients) for depression, 64% (27/42 patients) for bipolar disorder, 45% (10/22 patients) for psychophysiological insomnia, 41% (9/21 patients) for schizophrenia, 50% (7/14 patients) for anxiety disorders, 64% (7/11 patients) for epilepsy, 40% (4/10 patients) for adjustment disorders, and 80% (8/10 patients) for substance dependence. Compared with the non‐reduced group, significantly more patients with bipolar disorder and mood disorders involving depression in the reduced group had their prescriptions reduced to two or fewer drugs.

#### 3.3.3 Psychotropic drugs added within 1 year

Regarding sleep medications by psychotropic drug added within 1 year, the highest the reduction rate to two drugs or fewer was 86% (12/14 patients) in the trazodone addition group, followed by 77% (10/13 patients) in the chlorpromazine addition group, 70% (7/10 patients) in the aripiprazole addition group, and 61% (14/23 patients) in the quetiapine addition group.

On the other hand, the lowest reduction rate to two drugs or fewer was 27% (3/11 patients) in the suvorexant addition group, followed by 29% (2/7 patients) in the flunitrazepam addition group, 33% (4/12 patients) in the eszopiclone addition group, 42% (5/12 patients) in the olanzapine addition group, and 44% (4/9 patients) in the etizolam addition group.

#### 3.3.4 Factors involved in a successful hypnotic drug reduction

In a univariate logistic regression analysis conducted between the reduction and non‐reduction groups on sleeping pills within 1 year, significantly associations were found with age (mean ± SD: reduction group = 52.1 ± 15.8 years vs. non‐reduction group = 44.3 ± 16.4 years), the addition of trazodone [reduction group (*n* = 12) vs. non‐reduction group (*n* = 2)], and number of years of psychiatric experience (mean ± SD: reduction group = 14.4 ± 6.2 years vs. non‐reduction group = 12.3 ± 8.1 years). To adjust for confounding, multivariate logistic regression analysis was performed for “age,” “bipolar disorder,” “substance dependence,” “addition of trazodone,” “addition of chlorpromazine,” and “number of years of psychiatric experience,” the *p* values for all of which were <0.2 in the univariate logistic regression analysis.^8^ The results showed that the items with significant differences were age (every 1‐year increase: OR: 0.97, *p* < 0.0037; 95%CI: 0.95–0.99), the addition of trazodone (OR: 12.86, *p* < 0.0194; 95%CI: 1.511–109.4), and number of years of psychiatric experience (OR: 1.046, *p* < 0.00371; 95%CI: 1.003–1091).

From Table 2, we determined potential confounding factors (age, gender, mood disorder, anxiety disorder, and etizolam) for each of the factors that may be associated with discontinuation of multidrug use, based on medical considerations and previous studies.^10^, ^11^, ^12^, ^13^, ^14^, ^15^, ^16^, ^17^ The results were summarized in a Table S1 with adjusted for these factors. The results showed that “age,” “the addition of trazodone,” and “number of years of psychiatric experience” were still significantly associated factors after adjustment.

### 3.4 Percentage of hypnotic drugs that had been discontinued after 1 year by drug

**FIGURE 2:**
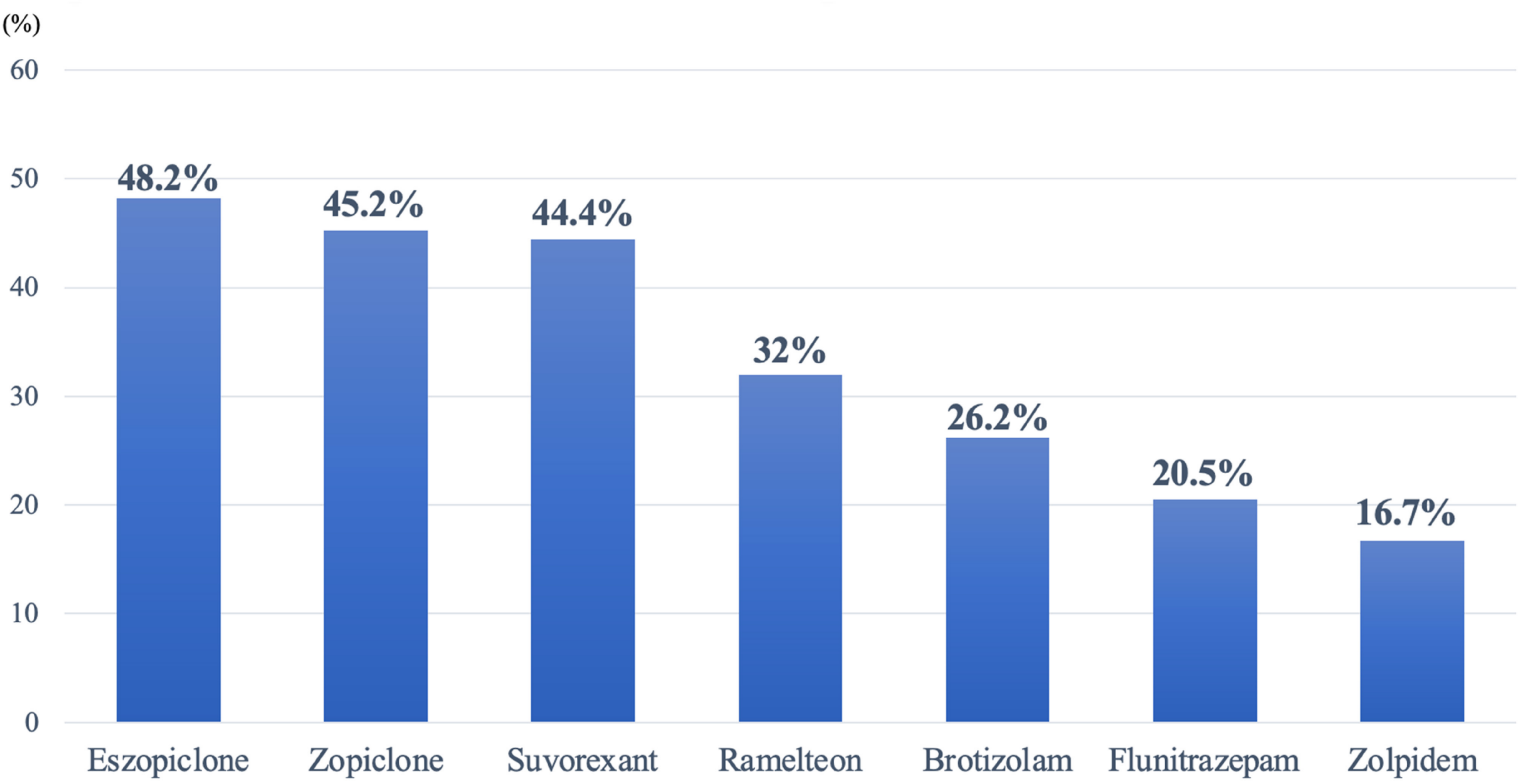
Discontinuation rate after 1 year for each hypnotic drug in outpatient psychiatry.

The percentage of hypnotic drugs that had been discontinued after 1 year was calculated for each of the multiple prescribed drugs (Figure 2). The hypnotic drug with the highest discontinuation rate after 1 year was eszopiclone (48.2%), followed by zopiclone (45.2%) and suvorexant (44.4%). By contrast, the hypnotic drug with the lowest rate of discontinuation after 1 year was zolpidem (16.7%), followed by flunitrazepam (20.5%) and brotizolam (26.2%).

### 3.5 Hypnotic drug reduction rate by the attending physician and number of years of psychiatric experience

**FIGURE 3:**
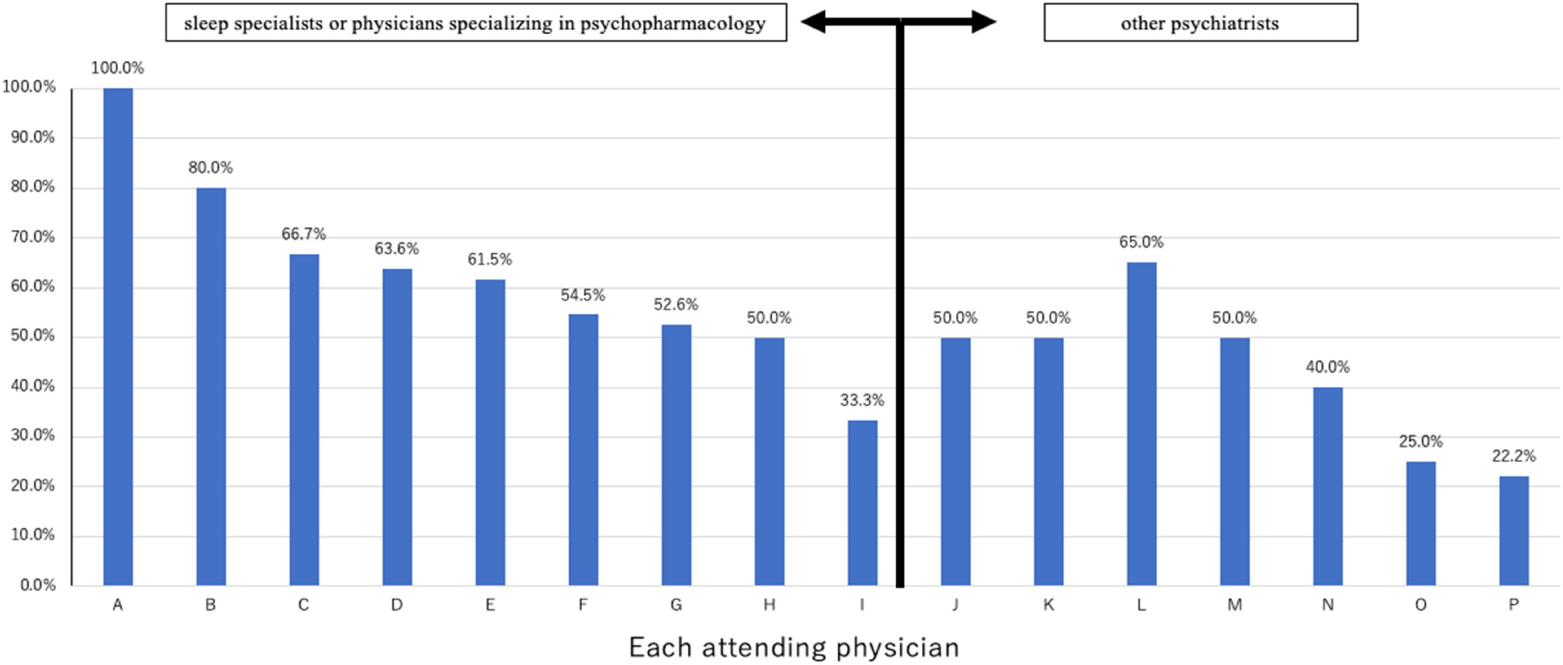
Rate of hypnotic drug reduction by each attending physician.

**FIGURE 4:**
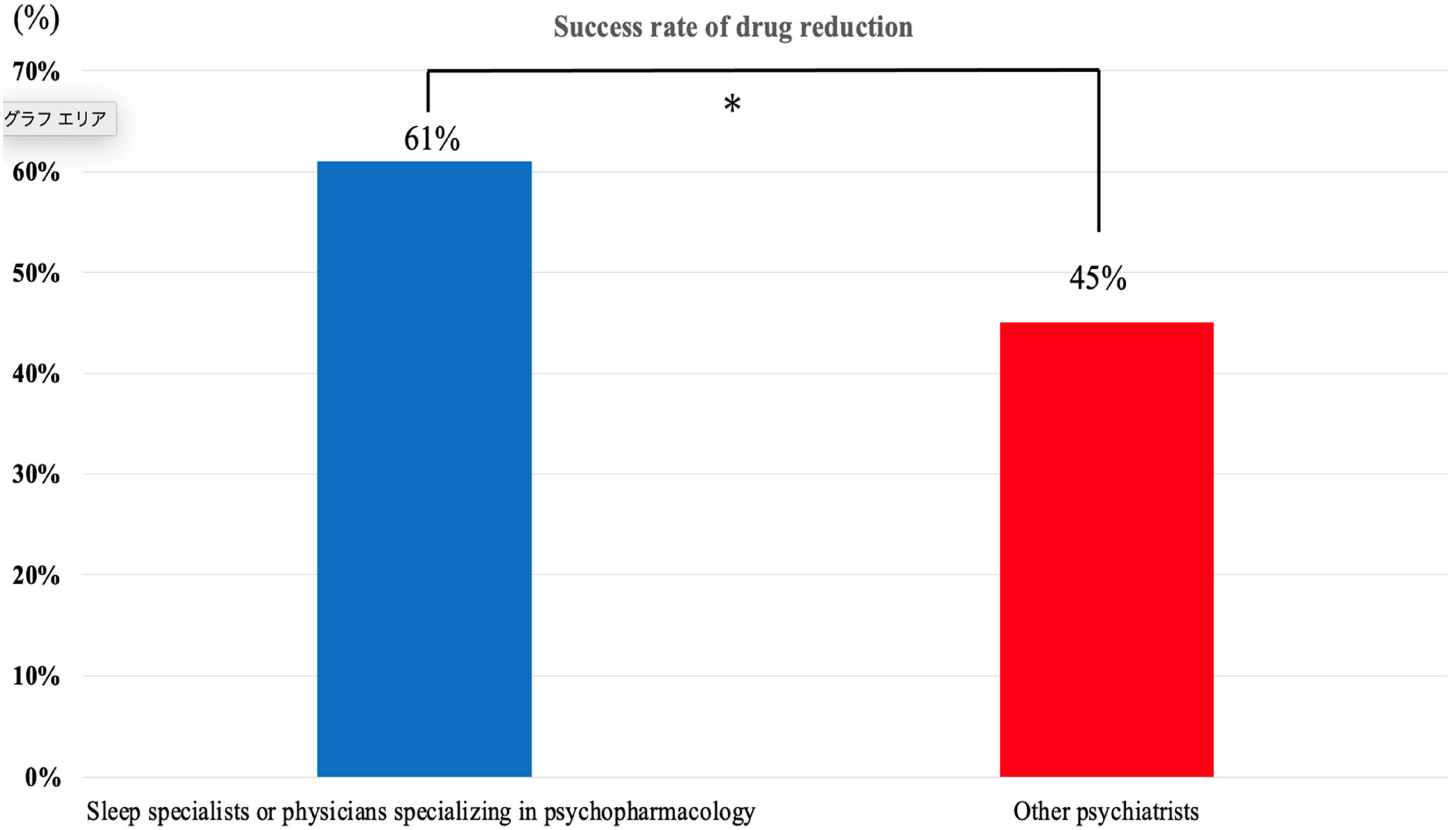
Success rate of reducing hypnotic drugs by years of experience and specialty. *Groups differed significantly at *p* < 0.05.

Figure 3 shows the hypnotic drug reduction rates after 1 year by the clinical physician who was in charge of the patient with multiple hypnotic drug prescriptions. The percentage of patients who had eliminated multiple prescriptions of hypnotic drugs after 1 year varied widely by attending physician, with a maximum of 100% (4/4 patients) and a minimum of 14.3% (1/7 patients). Figure 4 shows the hypnotic drug reduction rates after 1 year according to the attending physician by years of experience and specialty (“sleep specialist or physician specializing in psychopharmacology” and “other psychiatrists”). The rates of eliminating multidrug prescriptions of hypnotic drugs after 1 year were 61% for “sleep specialist or physician specializing in psychopharmacology” and 45% for “other psychiatrists.” The drug reduction success rate was significantly different between the two groups (*p* < 0.0316).

**FIGURE 5:**
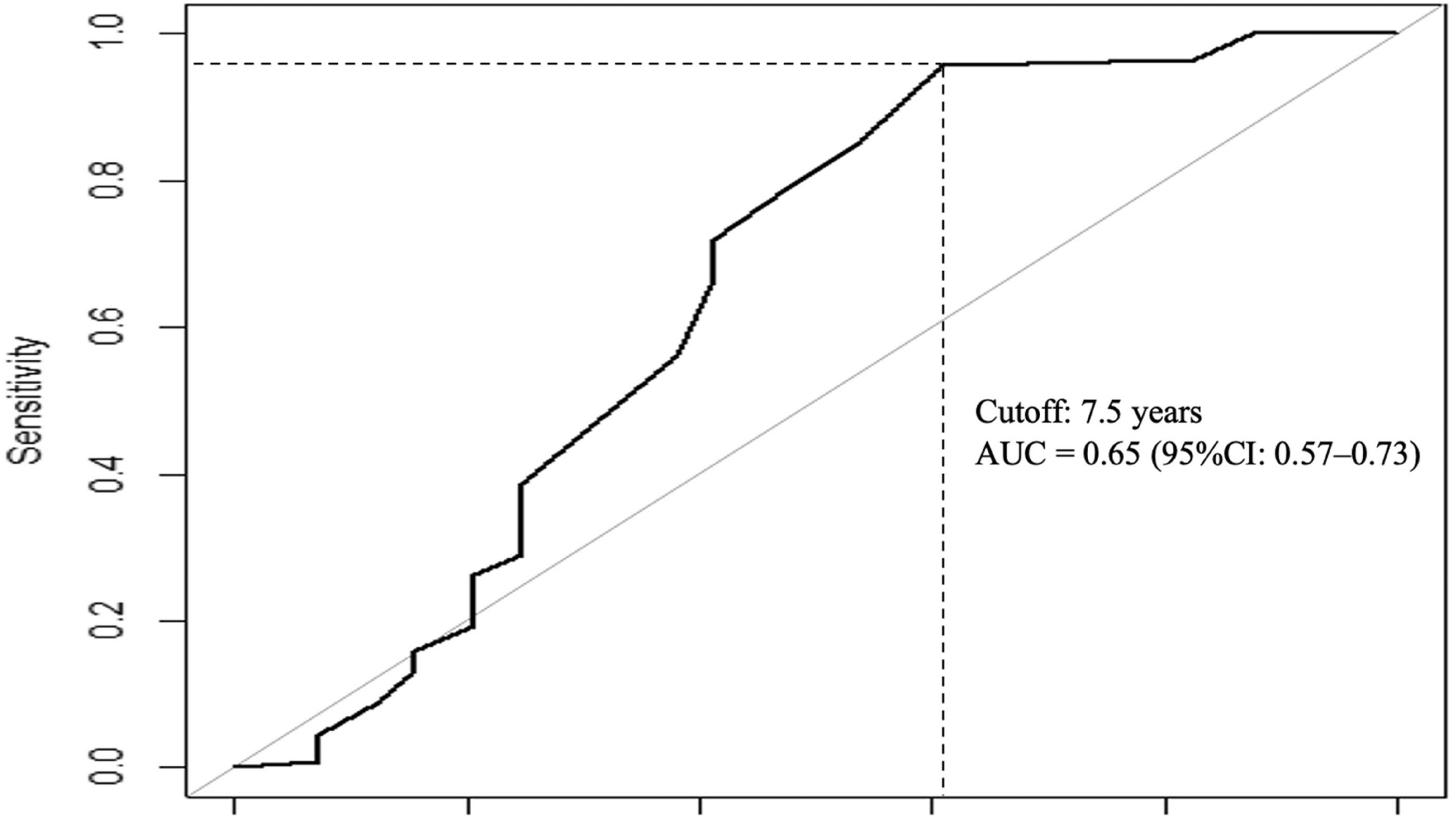
ROC curve analysis of the success rate of reducing hypnotic drugs by number of years of psychiatric experience. The AUC was 0.65, sensitivity 95.6%, and specificity 39.2% based on Youden's Index (intersection of dashed lines).

In addition, the cutoff values for the success rate of reducing hypnotic drugs by the number of years of psychiatric experience were explored using ROC analysis of Youden's Index. The results showed that the cutoff value was 7.5 years, with a sensitivity of 95.6% and a specificity of 39.2% (Figure 5).

## 4 DISCUSSION

### 4.1 Frequency of prescription of three or more hypnotic drugs

In this study, the frequency of prescribing three or more hypnotic drugs in our psychiatry department was about 6 times higher than that in other departments (excluding the pediatric department) when compared with the number of prescriptions by month. This result was similar to that reported by Mishima et al.^6^ who found that the frequency of use of multiple hypnotic drugs in psychiatry (9.8%–11.3%) was about 6–10 times higher than that in general medicine (1.3%–2.1%) during 2005–2009, indicating a tendency among patients with psychiatric disorders to receive prescriptions for multiple drugs. A previous study of the deterrent effect of the revision of medical fees on the concomitant use of hypnotics reported that the rate of concomitant use of hypnotics was not affected after the 2014 revision (from 5% to 4.8% in 1 year), but a decreasing trend was observed after the 2016 revision (from 4.7% to 3.7%).^18^ However, few reports have discussed these subsequent changes. According to the present study, from 2013 to 2019, the multidrug prescription rate for hypnotic drugs decreased from about 6% to 3.6% in psychiatric outpatients and from about 0.7% to 0.4% in general departments, indicating that revisions to medical fees may have had a substantial impact on the prevention of multidrug prescriptions afterwards.

### 4.2 Success factors for reducing multiple prescriptions of hypnotic drugs

Regarding the examination of factors related to the success or absence of a reduction in three or more drug prescriptions, the mean age ± SD of the reduction group was 52.1 ± 15.8 years, while that of the non‐reduction group was 44.3 ± 16.4 years; this significant difference was also recognized in the factorial analysis. When considering the frequency of hypnotic drug prescriptions, it is important to look at the extent to which polypharmacy is continued, as well as the characteristics of the patient population prone to polypharmacy, because a longer period of prescribing three drugs leads to an increase in the rate of prescribing multiple drugs. In a previous study, Arakawa et al.^19^ reported that the prescription rate of hypnotic drugs in psychiatry and psychosomatic medicine was higher among young to middle‐aged patients in their 20s to 50s than among those aged 65 years and older. The results of the present study also suggest that a reduction in the prescription rate for hypnotic drugs is less likely to be achieved in younger patients, which may be related to the high rate of multiple prescriptions among younger patients. Furthermore, the administration of multiple hypnotic drugs, such as benzodiazepines, to older adults has been shown to increase the risk of falls and fractures.^20^, ^21^ Therefore, older adults are often in a situation where hypnotic drugs must be reduced for adverse effects, whereas this is less likely to be the case in younger patients. In other words, it could be speculated that the difference in those incidences may have contributed to our finding that medications are less likely to be reduced in younger patients.

In addition, our results also indicated that the addition of trazodone was a significant success factor for drug reduction (*p* < 0.0194; 95%CI: 1.511–109.4). Trazodone is a sedating antidepressant widely used in clinical practice to treat insomnia and is the second most commonly prescribed drug for insomnia treatment in the United States after non‐benzodiazepine drugs.^22^ Trazodone is also prescribed in many insomnia treatment settings in Japan. A systematic review^23^ of trazodone in 2017 showed that the effect of improving total sleep duration for insomnia was greater than that of quetiapine.^24^ In addition, the efficacy of trazodone in reducing the dose of medication after 5 weeks among benzodiazepine‐dependent patients and as an alternative to benzodiazepines has also been reported.^25^, ^26^ In accordance with these previous studies, the present results also suggest that the addition of trazodone to patients with more than three prescriptions may promote success in reducing the number of drug prescriptions. However, even in previous studies, only reports of the effectiveness over the short term and in small doses are available; the effectiveness and safety of long‐term administration have not yet been established. In addition, in the present study, the starting dose of trazodone, changes in trazodone oral administration, and duration of oral administration (e.g., long‐term, short‐term) were investigated in detail. These factors need to be reevaluated in additional investigations in the future.

### 4.3 For discontinuation rates at 1 year by each hypnotic drug

In the present study, the lowest discontinuation rate among all hypnotic drugs after 1 year was 16.7% for zolpidem, an ultra‐short‐acting sleep medication. The Japanese guidelines for drug withdrawal recommend that patients taking multiple hypnotic drugs should reduce their dosages over a longer period of time compared with those taking a single hypnotic drug, taking rebound insomnia, tolerance formation, and withdrawal symptoms into account, and should start with hypnotic drugs with a shorter half‐life. It has also been reported that benzodiazepines with short blood half‐lives and high potencies tend to cause withdrawal symptoms and be difficult to discontinue.^25^, ^26^ In addition, in terms of the pharmacological mechanisms of action, zolpidem is known to have a particularly higher affinity for α_1_ subunits of the GABA_A_ receptor α subunit and to be more closely associated with drug‐dependent formation than non‐benzodiazepine hypnotics, such as eszopiclone and zopiclone.^27^, ^28^ Therefore, our results indicate that zolpidem may be a sleep medication that is clinically more difficult to discontinue, which suggests that zolpidem prescriptions are a factor in the difficulties associated with the amelioration of multiple drug prescribing situations in patients at risk of developing multiple drug use in the future, and as such, should be administered with caution when prescribed.

### 4.4 Drug reduction rate by attending physician and number of years of psychiatric experience

The results of this study indicated that although the elimination of multiple prescriptions after 1 year varied by attending physicians, sleep and pharmacology specialists had the highest rate of elimination of multiple prescription. In addition, 7.5 years was identified as the most successful amount of psychiatric experience for reducing multiple prescriptions of hypnotic drugs. However, it is difficult to discuss these results because of the lack of previous studies. In one of the few previous studies, the “Survey of the Actual Conditions of Drug‐related Psychiatric Disorders in Psychiatric Medical Facilities throughout Japan”^29^ reported that the majority of prescriptions (60.0%) were made by “psychiatrists,” 15.0% by “physicians in both psychiatry and physical departments,” and 7.1% by “physicians” for patients with sleeping/anxiolytic drug‐related disorders, and that many of these patients developed issues in relation to abuse and dependence during treatment for comorbid psychiatric disorders.^30^ A similar trend was found in a previous study by Matsumoto et al.^31^ who pointed out the existence of problems such as “rambling prescribing” by some psychiatrists, as well as “multiple drug combination therapy and mass therapy,” and “prescribing without consultation.” Although we were unable to examine each attending physician's patient population in detail in the present survey, the fact that the rate of elimination of multiple drug use was higher among attending physicians who were specializing in psychopharmacology clinical research and sleep medicine suggests that a thorough understanding of drug characteristics and attitudes regarding proceeding with treatment with an eye toward a way out may also have a small influence on whether a patient would reduce his or her medication.

As previously mentioned, it is difficult to discuss the years of psychiatric experience because of the lack of studies. However, the curriculum for psychiatrists involves learning general psychiatry within 5 years. After that, the curriculum is designed to enable the mastery of subspecialties such as sleep research and pharmacology over the next 5 years. The results of the present study regarding the effectiveness of 7.5 years of psychiatric experience coincide with the period during which subspecialties are learned. In other words, experience as a psychiatrist with expertise in the field was thought to lead to a better understanding of drug characteristics and increase the likelihood of successful drug reduction.

### 4.5 Limitations

This study had several limitations. First, as this was a retrospective study, the results should be interpreted with caution. Second, sleep problems were only screened for, not diagnosed; no objective measures of sleep or data from sleep diaries were used. Third, in this study, we did not consider the dose of drugs, severity and duration of mental illness, or the presence of any physical disease that could cause a sleep disorder (e.g., severe obesity, restless legs syndrome, sleep apnea syndrome). Fourth, the demographic data used in this study did not include body mass index, education period, exercise, smoking, or drinking habits. Fifth, because the study was conducted at a single institution, there is a possibility of subject selection bias by region or institution. Sixth, the adjusted analysis in this study is only a diversion of factors associated with clinical and “sleep medication monotherapy” reductions. Finally, the duration of insomnia, the duration of hypnotic drug use, and the details of each attending physician's patient population could not be assessed. However, despite these limitations, the present study is one of the few to examine the factors that reduce the number of multidrug prescriptions. Further research is needed to rule out the effects of these limitations.

## 5 CONCLUSION

The revisions for psychotropic medications, including sleeping pills, made after 2014 had some effect on the elimination of multiple prescriptions of sleeping pills in our hospital. This finding highlights the importance of planning for a future reduction in dosage or discontinuation from the beginning of treatment, and this was considered to be even more important in the treatment of insomnia, especially for younger patients, who tend to have difficulty reducing their dosage of sleeping pills. The use of trazodone as an alternative therapy to hypnotic drugs is controversial, but our findings suggest that it may provide a clue to solving the polypharmacy problem of hypnotic drugs, at least for short‐term administration aimed at reducing the dosage. Above all, it is important that as many therapists as possible recognize the importance of advancing treatment with solutions for the multidrug prescription drug problem in mind.

## AUTHOR CONTRIBUTIONS

Takao Kato and Nozomu Kotorii conceived and designed the study. Motohiro Ozone and Naohisa Uchimura supervised the study. Takao Kato, Nozomu Kotorii, Hayato Ohshima, Hiroyuki Mori, Kenjirou Wasano, Hiroshi Hiejima, and Mitsunari Habukawa collected the data. Takao Kato, Nozomu Kotorii, and Kenta Murotani analyzed the data. Takao Kato and Nozomu Kotorii drafted the manuscript. All authors provided critical comments on the manuscript.

## FUNDING INFORMATION

The authors have received no financial support for the research, writing, and/or publication of this paper.

## CONFLICT OF INTEREST STATEMENT

The authors declare no conflict of interest.

## ETHICS STATEMENT

*Approval of the Research Protocol by an Institutional Reviewer Board*: This study complied with the ethical principles laid down in the Declaration of Helsinki and was conducted in accordance with the “Ethical Guidelines for Medical Research Involving Human Subjects.” This study was approved by the Ethics Committee of Kurume University (No. 19272).

*Informed consent*: The requirement for informed consent was waived by our ethics committee because of the retrospective nature of the study.

*Registry and the registration no. of the study/trial*: N/A.

*Animal studies*: N/A.

## Supporting information


Table S1.

## Data Availability

We did not obtain informed consent for the release of our data and therefore cannot share the data with a third party.

## References

[1] Buysse DJ . Chronic insomnia. Am J Psychiatry. 2008;165:678–686. 18519533 10.1176/appi.ajp.2008.08010129 PMC2859710

[2] Pigeon WR , Perlis ML . Sleep homeostasis in primary insomnia. Sleep Med Rev. 2006;10:247–254. 16563817 10.1016/j.smrv.2005.09.002

[3] Cappuccio FP , D'Elia L , Strazzullo P , Miller MA . Quantity and quality of sleep and incidence of type 2 diabetes: a systematic review and meta‐analysis. Diabetes Care. 2010;33:414–420. 19910503 10.2337/dc09-1124 PMC2809295

[4] Spiegel K , Leproult R , Van Cauter E . Impact of sleep debt on metabolic and endocrine function. Lancet. 1999;354:1435–1439. 10543671 10.1016/S0140-6736(99)01376-8

[5] Baglioni C , Battagliese G , Feige B , Spiegelhalder K , Nissen C , Voderholzer U , et al. Insomnia as a predictor of depression: a meta‐analytic evaluation of longitudinal epidemiological studies. J Affect Disord. 2011;135:10–19. 21300408 10.1016/j.jad.2011.01.011

[6] Mishima K , Riemann D . A survey study on psychotropics prescription using medical fee data. Health and Labor Sciences Research Grant and Health and Labor Sciences Special Research Project “Comparative Research on the Prescription Status of Psychotropic Drugs in Japan and Overseas” 2010 Research Report. 2011, pp. 15–32.

[7] Okumura Y , Inada K , Matsumoto T . Clinical psychopharmacology of changes in high‐dose and multi‐drug prescriptions of antianxiety drugs and hypnotics due to revision of medical treatment fees. J Clin Psychopharmacol. 2015;18:11173–11188.

[8] Katz MH . Multivariable analysis. A practical guide for clinicians second edition. Cambridge: Cambridge University Press; 2006.

[9] R Core Team . R: a language and environment for statistical computing. Vienna, Austria: R Foundation for Statistical Computing; 2021.

[10] Rickels K , Case WG , Schweizer EE , Garcia F , Fridman R . Long‐term benzodiazepine users 3 years after participation in a discontinuation program. Am J Psychiatry. 1991;148:757–761. 2035717 10.1176/ajp.148.6.757

[11] Holton A , Riley P , Tyrer P . Factors predicting long‐term outcome after chronic benzodiazepine therapy. J Affect Disord. 1992;24:245–252. 1578080 10.1016/0165-0327(92)90109-j

[12] Rickels K , Schweizer E , Case WG , Greenblatt DJ . Long‐term therapeutic of benzodiazepines. I. Effects of abrupt discontinuation. Arch Gen Psychiatry. 1990;47:899–907. 2222129 10.1001/archpsyc.1990.01810220015002

[13] Schweizer E , Rickels R , De Martinis N , Case G , Garcia‐Espana F . The effect of personality on withdrawal severity and taper outcome in benzodiazepine dependent patients. Psychol Med. 1998;28:713–720. 9626727 10.1017/s0033291798006540

[14] Rickels K , DeMartinis N , Garcia‐Espana F , Greenblat DJ , Mandos LA , Rynn M . Imipramine and buspirone in treatment of patients with generalized anxiety disorder who are discontinuing long‐term benzodiazepine therapy. Am J Psychiatry. 2000;157:1973–1979. 11097963 10.1176/appi.ajp.157.12.1973

[15] Murphy SM , Tyrer P . A double‐blind comparison of the effects of gradual withdrawal of lorazepam, diazepam and bromazepam in benzodiazepine dependence. Br J Psychiatry. 1991;158:511–516. 1675901 10.1192/bjp.158.4.511

[16] Ashton H . Benzodiazepine withdrawal: outcome in 50 patients. Br J Addict. 1987;82:665–671. 2886145 10.1111/j.1360-0443.1987.tb01529.x

[17] Golombok S , Higgitt A , Fonagy P , Dodds S , Saper J , Lader M . A follow‐up study of patients treated for benzodiazepine dependence. Br J Med Psychol. 1987;60:141–149. 2887197 10.1111/j.2044-8341.1987.tb02724.x

[18] Mishima K . FY2017‐29 Comprehensive Research Project on Policies for Persons with Disabilities, “Research on Guidelines for Pharmacotherapy to Elucidate the Psychotropic Drug Prescription and Practice Appropriate Prescription (H291 Psychiatry 1101)” Shared Research Report. 2017.

[19] Arakawa R . Investigation of prescription status of antianxiety drugs and hypnotics in outpatient care using a national database. Clin Psychiat. 2015;44:1003–1010.

[20] Holbrook AM , Crowther R , Lotter A , Cheng C , King D . Meta‐analysis of benzodiazepine use in the treatment of insomnia. CMAJ. 2000;162:225–233. 10674059 PMC1232276

[21] Glass J , Lanctôt KL , Herrmann N , Sproule BA , Busto UE . Sedative hypnotics in older people with insomnia: meta‐analysis of risks and benefits. BMJ. 2005;331:1169. 16284208 10.1136/bmj.38623.768588.47 PMC1285093

[22] Bertisch SM , Herzig SJ , Winkelman JW , Buettner C . National use of prescription medications for insomnia: NHANES 1999–2010. Sleep. 2014;37:343–349. 24497662 10.5665/sleep.3410 PMC3900622

[23] Jaffer YK , Chang T , Vanle B , Dang J , Steiner AJ , Loera N , et al. Trazodone for insomnia: a systematic review. Innov Clin Neurosci. 2017;14:24–34. PMC5842888 29552421

[24] Doroudgar S , Chou TI , Yu J , Trinh K , Pal J , Perry PJ . Evaluation of trazodone and quetiapine for insomnia: an observational study in psychiatric inpatients. Prim Care Companion CNS Disord. 2013;15:13m01558. 10.4088/PCC.13m01558 PMC3977773 24800124

[25] Rickels K , Case WG , Schweizer EE , Swenson C , Fridman RB . Low‐dose dependence in chronic benzodiazepine users: a preliminary report on 119 patients. Psychopharmacol Bull. 1986;22:407–415. 2877472

[26] Hallfors DD , Saxe L . The dependence potential of short half‐life benzodiazepines: a meta‐analysis. Am J Public Health. 1993;83:1300–1304. 8103297 10.2105/ajph.83.9.1300 PMC1694983

[27] Nutt DJ , Stahl SM . Search for perfect sleep: the continuing evolution of GABAA receptor modulators as hypnotics. J Psychopharmacol. 2010;24:1601–1612. 19942638 10.1177/0269881109106927

[28] Frankle WG , Cho RY , Prasad KM , Mason NS , Paris J , Himes ML , et al. In vivo measurement of GABA transmission in healthy subjects and schizophrenia patients. Am J Psychiatry. 2015;172:1159–1178. 10.1176/appi.ajp.2015.14081031 PMC5070491 26133962

[29] Matsumoto T , Ozaki S , Kobayashi S , Wada K . Current situation and clinical characteristics of sedative‐related disorder patients in Japan: a comparison with methamphetamine‐related disorder patients. Psychiatr Mag. 2011;113:1184–1198. 22352004

[30] Matsumoto T , Ito T , Takano T . A survey of the actual conditions of drug‐related psychiatric disorders in psychiatric care facilities nationwide. FY2016 Health, Labour and Welfare Science Research Grant (Research Report on Regulatory Science Policy Research Projects for Pharmaceuticals and Medical Devices). 2017.

[31] Matsumoto T , Naruse N , Umeno M . A study on the clinical characteristics of benzodiazepine use disorders and the characteristics of psychiatric treatment that triggered the onset. J Jpn Soc Alc Pharm Med. 2012;47:317–330. 23461220

