# Supplementary material for: Trends in the multiple prescriptions of hypnotic drugs in a university outpatient in Japan
Source: Neuropsychopharmacol Rep. 2023 Nov 9;44(1):80–9. doi: 10.1002/npr2.12386 (PMC10932787; doi:10.1002/npr2.12386)
Supplement: Supplementary file 1 — Table S1. [file NPR2-44-80-s001.zip › supplemental table 2.docx]

**SUPPLEMENTAL TABLE**

*groups differed significantly at p < 0.05.

Abbreviations: SD, standard deviation; CI, confidence interval.

†adjusted for age, gender, hyperbolic affective disorder, etizolam use, depression, anxiety disorder.
